# The heptad repeat region is a major selection target in MERS-CoV and related coronaviruses

**DOI:** 10.1038/srep14480

**Published:** 2015-09-25

**Authors:** Diego Forni, Giulia Filippi, Rachele Cagliani, Luca De Gioia, Uberto Pozzoli, Nasser Al-Daghri, Mario Clerici, Manuela Sironi

**Affiliations:** 1 Scientific Institute IRCCS E. MEDEA, Bioinformatics, 23842 Bosisio Parini, Italy; 2 Department of Biotechnology and Biosciences, University of Milan-Bicocca, 20126 Milan, Italy; 3 Biomarkers research program, Biochemistry Department, College of Science, King Saud University, Riyadh 11451, Kingdom of Saudi Arabia (KSA); 4 Prince Mutaib Chair for Biomarkers of Osteoporosis, Biochemistry Department, College of science, King Saud University, Riyadh, KSA; 5 Department of Physiopathology and Transplantation, University of Milan, 20090 Milan, Italy; 6 Don C. Gnocchi Foundation ONLUS, IRCCS, 20148 Milan, Italy

## Abstract

Middle East respiratory syndrome coronavirus (MERS-CoV) originated in bats and spread to humans via zoonotic transmission from camels. We analyzed the evolution of the *spike* (*S*) gene in betacoronaviruses (betaCoVs) isolated from different mammals, in bat coronavirus populations, as well as in MERS-CoV strains from the current outbreak. Results indicated several positively selected sites located in the region comprising the two heptad repeats (HR1 and HR2) and their linker. Two sites (R652 and V1060) were positively selected in the betaCoVs phylogeny and correspond to mutations associated with expanded host range in other coronaviruses. During the most recent evolution of MERS-CoV, adaptive mutations in the HR1 (Q/R/H1020) arose in camels or in a previous host and spread to humans. We determined that different residues at position 1020 establish distinct inter- and intra-helical interactions and affect the stability of the six-helix bundle formed by the HRs. A similar effect on stability was observed for a nearby mutation (T1015N) that increases MERS-CoV infection efficiency *in vitro*. Data herein indicate that the heptad repeat region was a major target of adaptive evolution in MERS-CoV-related viruses; these results are relevant for the design of fusion inhibitor peptides with antiviral function.

Middle East respiratory syndrome coronavirus (MERS-CoV), a newly emerged virus that can cause severe lower respiratory tract infection in humans, was first identified in Saudi Arabia in 2012^1^. Since then, 945 laboratory-confirmed cases of MERS-CoV infection, leading to at least 348 related deaths, have been reported to the WHO (as of January 5^th^, 2015) (http://www.who.int/csr/don/05-january-2015-mers-jordan/en/).

MERS-CoV belongs to the clade c of betacoronaviruses (betaCoVs)^2^, which also includes two bat sister species, namely Ty-BatCoV HKU4 and Pi-BatCoV HKU5, isolated from the lesser bamboo bats (*Tylonycteris pachypus*) and Japanese pipistrelles (*Pipistrellus abramus*), respectively^3^. Additional viruses related to MERS-CoV have been described in bats (BtCoV/KW2E-F93, BtCoV/133) and hedgehogs (*Erinaceus* coronavirus, EriCoV)^4^,^5^. Recently, a virus belonging to the same species as MERS-CoV was isolated in *Neoromicia* bats (NeoCoV), supporting a bat-origin for MERS-CoV^6^. This hypothesis received further confirmation by the identification of dipeptidyl-peptidase 4 (DPP4, also known as CD26) as the cellular receptor used by both Ty-BatCoV HKU4 and MERS-CoV^7^,^8^,^9^.

Notably, high titers of MERS-CoV neutralizing antibodies have been detected in camels from various countries and MERS-CoV has been isolated from these animals in Saudi Arabia and Qatar^10^. Genetic diversity is slightly higher for camel-derived viruses compared to human MERS-CoV isolates, suggesting camel to human transmission rather than vice versa^10^. Therefore, the most likely scenario envisages that a bat-derived MERS-CoV spread to humans via the zoonotic transmission from dromedary camels.

Coronaviruses use their spike (S) protein to bind a host receptor and to promote membrane fusion. The spike protein assembles as a trimer on the viral surface and belongs to the class I fusion protein family^11^. Class I fusion proteins are found in many other virus genera including retroviruses, orthomyxoviruses, paramyxoviruses, and filoviruses and share similar domain organization, as well as common functional properties^12^.

Host proteases cleave the CoV spike protein into two functionally distinct domains: the N-terminal region (usually referred to as the S1 subunit) contains the receptor binding domain (RBD), whereas the C-terminal portion (S2 subunit) includes the fusion peptide, two heptad repeats (HR1 and HR2), and the transmembrane (TM) domain^12^ (see Fig. 1A). Following receptor binding, membrane fusion is mediated by a major conformational rearrangement that exposes the fusion peptide and results in the formation of a six-helix bundle (6HB)^13^,^14^. The core of the 6HB is a triple-stranded coiled coil formed by the HR1s of the three spike subunits forming the trimer; the HR2 elements pack within the grooves of the coiled coil in an antiparallel direction^13^,^14^.

Because of its central role in membrane fusion, a number of antiviral peptides that interfere with the 6HB formation have been developed as potential therapeutic compounds against HIV^15^, Ebola virus^16^, SARS-CoV^17^,^18^, and MERS-CoV^14^.

Although the RBD of spike proteins is generally considered the major determinant of host range, several reports have suggested that variation in the C-terminal portion of spike proteins, particularly in the HR1 and HR2, determine host range expansion^12^. Moreover, recent works indicated that MERS-CoV and Ty-BatCoV HKU4 bind DPP4 both of human and of bat origin^7^,^8^,^9^. In particular, although MERS-CoV binds human DPP4 with higher affinity than Ty-BatCoV HKU4, which shows a preference for the bat receptor, the RBDs of the two viruses engage human DPP4 via a similar binding mode^9^. These observations suggest that MERS-CoV and related viruses have the potential to shift host range with little adaptation of the RBD.

Motivated by the notion that evolutionary analyses can provide information on the molecular events that underlie host shifts and, more generally, host-pathogen interactions^19^, we investigated the evolutionary history of S proteins in MERS-CoV and related betaCoVs. Specifically, we aimed to determine whether natural selection drove the evolution of specific regions and sites that may contribute to variation in host range or replication efficiency. Thus, using different strategies, we analyzed MERS-CoV strains isolated from human and camels, as well as MERS-CoV-related viruses from other mammals. Data indicate the HR1 to HR2 region as a major target of adaptive evolution in these viruses.

## Results

### Positive selection shaped the evolution of clade c betaCoV spike protein

**Figure 1:**
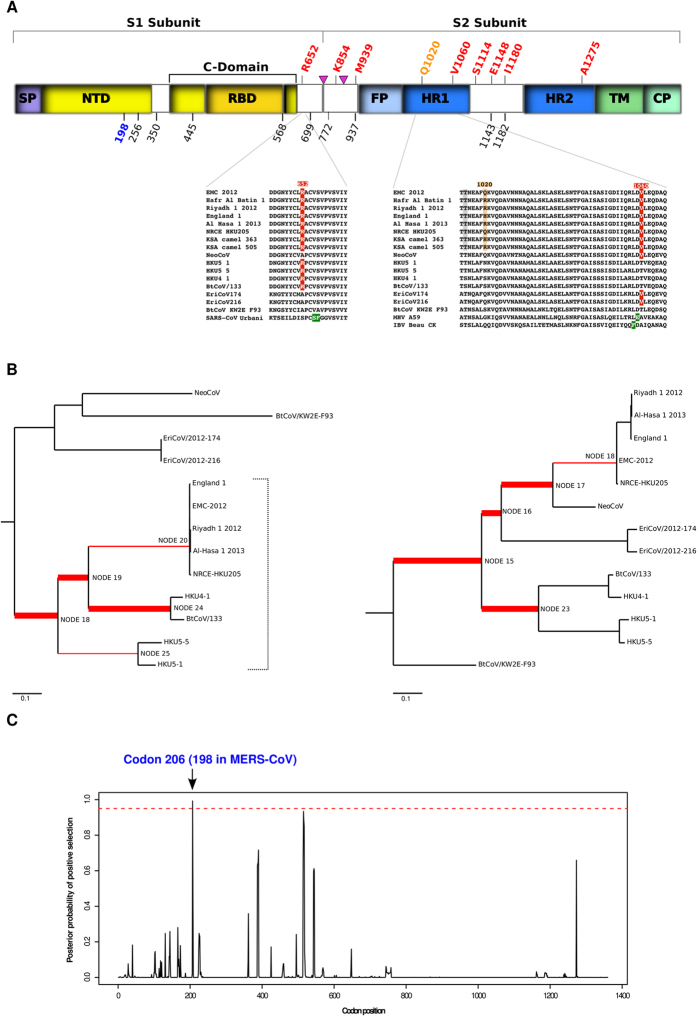
Positive selection at the *spike* gene of MERS-CoV-related coronaviruses. (**A**) Cartoon representation of the MERS-CoV spike protein with distinct domains in different colors (SP, signal peptide; NTD, N-terminal domain; RBD, receptor binding domain; FP, fusion peptide, HR1 and HR2, heptad repeat 1 and 2; TM, transmembrane domain; CP, cytoplasmic domain). The location of positively selected sites detected in MERS-CoV related sequences is shown in red. A positively selected residue in MERS-CoV isolated from humans and camels is in orange. The positively selected site and recombination breakpoints in Pi-BatCoV HKU5 sequences are shown in blue and black, respectively. Furin cleavage sites are depicted as triangles^56^. Two alignment portions are shown; positions that alter virus host range or tropism in SARS-CoV, MHV, and Beaudette strain IBV (IBV Beau CK) are highlighted in green^25^,^26^,^27^. A functional mutation which arose during tissue-culture adaptation of MERS-CoV (strain EMC2012) is highlighted in grey^31^. (**B**) Bayesian phylogenies for the S1 (left) and S2 (right) sequences^6^; branch length is proportional to dS. Branches in red were set as foreground lineages in independent branch-site tests. Thick branches yielded statistically significant evidence of positive selection. The bracket denotes a subset of sequences that were used for analysis of positive selection in the RBD. (**C**) OmegaMap results for Pi-BatCoV HKU5 *spike* genes. The hatched red line corresponds to a posterior probability of selection equal to 0.95.

We first investigated whether positive selection drove the evolution of MERS-CoV-related coronavirus spike proteins. Previous phylogenetic analyses of *S* genes of viruses isolated from humans/camels (MERS-CoV), bats, and hedgehogs (Supplementary Table. S1) indicated that the S1 and S2 regions display different tree topologies (Fig. 1B), possibly as a result of recombination^6^. Because recombination can inflate estimates of positive selection^20^, we separately analyzed the two regions.

We pruned the S1 and S2 multiple sequence alignments (MSAs) from unreliably aligned codons and we screened them for evidence of additional recombination events using GARD (Genetic Algorithm Recombination Detection)^21^, which detected no breakpoint.

The saturation of substitution rates represents a major problem in the detection of positive selection among distantly related sequences. Computation of the nonsynonymous (dN) and synonymous (dS) substitution rates over whole phylogenies allows breaking of long branches, resulting in improved rate estimation. Thus, evidence of episodic positive selection was searched for by using the *codeml* branch-site test^22^, which is relatively insensitive to the saturation of substitution rates^23^. In the S1 and S2 regions, 3 and 4 branches yielded statistically significant evidence of positive selection under different codon frequency models (Fig. 1B, Table 1 and Supplementary Table S3). Positively selected sites along these branches were detected using the BEB (Bayes empirical Bayes) procedure and validated using the Mixed Effects Model of Evolution (MEME)^24^.

One positively selected site was found in the S1 region, 7 sites in the S2 subunit (Fig. 1A, Table 1). The R652 selected site (in S1) almost corresponds to two mutations that independently arose in the SARS-CoV *spike* gene as a result of *in vitro* adaptation of zoonotic strains to primate cells^25^ (Fig. 1A). In S2, most selected sites are located in the HR1, HR2, and in the intervening linker. Remarkably, position 1060 is the almost exact counterpart of aminoacid changes that expand the host range or cell-type tropism of infectious bronchiolitis virus (IBV, L857F) and murine hepatitis virus (MHV, E1035D) (Fig. 1A)^26^,^27^.

No positively selected site was found to be located in the RBD (Fig. 1A). Nevertheless, the pruning of unreliably aligned codons operated on the MSA left a minority of RBD sites available for analysis. We thus repeated the branch-site test on a subset of more closely related sequences (Fig. 1B). This procedure decreased divergence and pruning in the RBD and allowed analysis of most codons; even with this procedure, no positively selected site was detected (not shown).

### Minor effect of positive selection for the *S* genes of Ty-BatCoV HKU4 and Pi-BatCoV HKU5

Previous analysis of Ty-BatCoV HKU4 and Pi-BatCoV HKU5 viruses isolated in Hong Kong indicated that positive selection targeted the spike protein and particularly its S1 region^28^. Nevertheless, recombination was not accounted for in that analysis. We thus analyzed the sequence alignments of the Hong Kong isolates for the presence of recombination breakpoints using GARD. The algorithm detected 9 recombination breakpoints for the Pi-BatCoV HKU5 alignment (Fig. 1A) and none for Ty-BatCoV HKU4. The Ty-BatCoV HKU4 *spike* gene was therefore analyzed using the codeml site models, which test the hypothesis that a subset of codons evolve with dN/dS > 1. No evidence of positive selection was found, even using the relatively non-conservative model M7/model M8 comparison (Supplementary Table S4). As for the Pi-BatCoV HKU5 *spike* gene, rampant recombination prevents application of a similar approach. We thus resorted to the simultaneous estimation of selection and recombination using omegaMap^29^. This analysis confirmed high recombination along the whole gene (Supplementary Fig. S1) and detected a single positively selected site in the S1 region (Fig. 1C). The same site (codon 198, position relative to the MERS-CoV spike sequence) was also detected by MEME, which was run by incorporating the alternative phylogenies detected by GARD. Most of the previously reported selected sites^28^ were not detected using other methods that account for recombination (Supplementary Table S5). We thus consider that robust inference of positive selection in Pi-BatCoV HKU5 *spike* genes can only be made for position 198, which lies outside the RBD (Fig. 1A).

### Positive selection in the MERS-CoV heptad repeat 1

We next wished to determine whether positive selection occurred at the *spike* gene of MERS-CoV viruses circulating in the recent outbreak. A previous study suggested that positive selection drove the evolution of two codons in the MERS-CoV *spike* gene (positions 509 and 1020)^30^. Nevertheless, in that study only one method was used to infer selection and sequences isolated from camels were not included.

We thus retrieved 54 fully or almost fully *spike* sequences of MERS-CoV isolated from camels or humans (Supplementary Table S1). Alignments for the S1 and S2 regions were separately analyzed and screened for the presence of recombination. No breakpoint was detected and the *codeml* site models were applied. For the S2 region, two models of gene evolution that allow a class of codons to evolve with dN/dS > 1 (NSsite models M2a and M8) showed better fit to the data than the null models (NSsite models M1a and M7), strongly supporting the action of positive selection (Table 2, Supplementary Table S6). No evidence of selection was detected for the S1 portion (Table 2, Supplementary Table S6). In S2, both BEB and MEME detected one selected site: position 1020 in the HR1 (Fig. 1A). Interestingly, three different residues are observed at this site in both camel- and human-derived viruses (Fig. 1A). MERS-CoV is thought to have spread from camels to humans; the presence of the three alternative residues in viruses isolated from camels suggests that adaptive evolution at this site occurred prior to the infection of humans.

**Figure 2:**
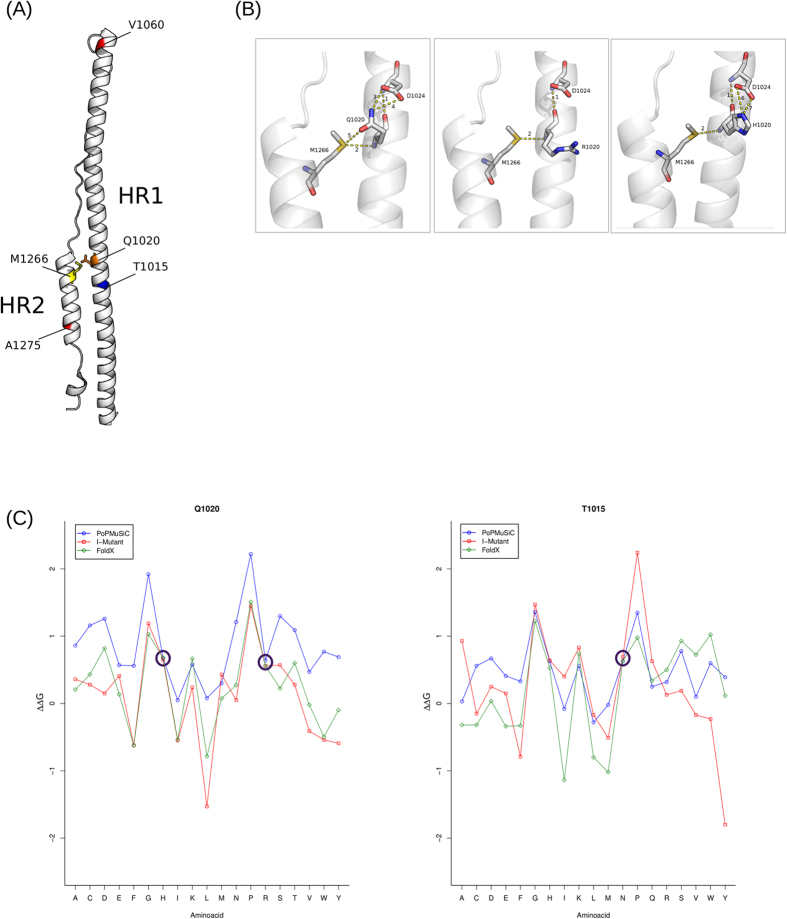
Analysis of variation in the MERS-CoV HR1. (**A**) Ribbon representation of MERS-CoV HR region. Positively selected sites in betaCoVs are shown in red; Q1020 (orange) and M1266 (yellow) are shown as sticks. T1015 is in blue. (**B**) Detail of inter- and intra-helical interactions for residue 1020. Interactions are shown for Q1020 (left), R1020 (middle) and H1020 (right). Hydrogen bonds are shown as hatched yellow lines; color codes: carbon, white; oxygen, red; nitrogen, blue; sulphur, yellow. Hydrogens have been omitted for clarity. (**C**) Stability analysis for HR1 positions 1020 (left) and 1015 (right). ΔΔG in kcal/mol for mutations of Q1020 and T1015 to all other 19 aminoacids are reported. Aminoacid residues observed in MERS-CoV sequences are circled. Results are shown for FoldX, PopMuSiC, and I-Mutant.

Interestingly, the 1020 variant is in proximity to a mutation (T1015N) (Figs 1A and 2A) that arose during tissue-culture adaptation of MERS-CoV (strain EMC2012) and increases replication efficiency^31^.

### Analysis of MERS-CoV HR1 variation

The structure of the 6HB of MERS-CoV has been solved^13^,^14^; through its side chain, the Q1020 residue forms hydrogen bonds with D1024 and interacts with M1266 in HR2 (Fig. 2A,B). Replacement of the glutamine residue with histidine or arginine (observed in the camel- and human-derived viruses) results in loss of side chain interactions with M1266 and variably affects hydrogen bonds with D1024 (Fig. 2B).

To gain further insight into the effect of adaptive evolution at position 1020, we performed a stability computational analysis after in silico mutagenesis. Q1020 was replaced with all other possible aminoacids: even if changes of different magnitude in ΔG were obtained using three different methods^32^,^33^,^34^, trends were very consistent (Fig. 2C). In particular, replacement with histidine or arginine residues resulted in mild destabilization (Fig. 2C). As a comparison, the same analysis was performed for position 1015. Replacement of the threonine residue with asparagine, which was previously associated with increased replication efficiency *in vitro*^31^, resulted in a similar level of destabilization as observed for Q1020H/R (Fig. 2C).

## Discussion

We analyzed the evolution of the S protein in betaCoVs, in bat Ty-BatCoV HKU4 and Pi-BatCoV HKU5 viral populations, as well as in MERS-CoV isolates from the current outbreak. Different strategies were applied, as appropriate depending on divergence and recombination.

Results indicated that several adaptive changes are located in the S2 region, with fewer in the S1 domain and none of these within the RBD. It should be noted, though, that in all analyses we applied quite conservative approaches and we intersected two different methods to declare a site as positively selected. Whereas this approach was meant to limit the false positive rate it may have yielded some false negative results. In particular, the branch-site test we used to analyze the S1 and S2 regions of betaCoVs is robust to saturation issues and has a minimal false positive rate, but lacks power^23^. Moreover, due to the high divergence and the consequent need of alignment pruning, analysis of the RBD was performed on a shallower phylogeny compared to the other regions. This procedure is expected to reduce power, but is nonetheless necessary. In fact, alignment errors, together with unrecognized recombination, inflate estimates of positive selection and represent major sources of false positive results in evolutionary analyses^20^,^35^. Consistently, when we accounted for recombination in Pi-BatCoV HKU5 sequences most previously described selection signals disappeared, including those in the RBD^28^. Thus, whereas we cannot exclude that adaptive variants in the RBD of betaCoVs were missed by our approach, we conclude that the more recent evolution of MERS-CoV, Ty-BatCoV HKU4, and Pi-BatCoV HKU5 was not driven by positive selection in this domain. Conversely, as previously shown for SARS-CoV^25^, our data support a role for the S1 region separating the RBD and fusion peptide as a determinant of betaCoV host range expansion.

Analysis of both betaCoVs and MERS-CoV strains revealed evidence of positive selection in the S2 region. Most positively selected sites were found to be located either in the heptad repeats or in the intervening linker. Among these sites, position 1060 is particularly interesting, as it almost corresponds to substitutions that modify the host range and/or cell tropism in MHV and IBV^26^,^27^. Both viruses belong to the *coronavirus* genus. The Beaudette strain of IBV has been adapted to embryonated chicken eggs; following passages in culture, the strain was further adapted to infect Vero cells (from African Green monkey) and primary chicken kidney cells^26^. The L857F mutation was shown to represent a major determinant of the fusogenic activity in these cell types^26^. As far as MHV is concerned, the E1035D mutation was recovered after passages in mouse liver and was shown to contribute significantly to the hepatotropism and hepatic virulence of a previously attenuated strain (MHV-A59)^27^. Additional variants in the HR1 and fusion peptide of MHV strains cooperate with changes in the S1 region, resulting in a broadening of receptor usage (to heparan sulfate) and, consequently, an extension of the host range^36^. Finally, in the MHV-A51 strain the ability to bind human CAECAM receptors is strongly influenced by mutant residues located in the fusion peptide, HR1 and HR1/HR2 linker^37^. Similar observations have been reported for viruses that do not belong to the *coronavirus* genus, but that use a class I fusion protein. For instance, one single mutation in the HR1 of a simian-human immunodeficiency virus (SHIV) strain (KB9) increases by two- to three-fold infection efficiency in cells expressing the marmoset cellular receptors^38^, whereas a nearby HR1 change in SIV contributes to macrophage tropism^39^. Overall, these findings pinpoint the relevance of changes in the HR1 and HR2 as modifiers of host range and cell-type tropism.

The molecular mechanisms underlying the altered phenotype of HR1 and HR2 mutants remain to be determined in all these instances, although changes in conformational structure have been suggested as a possible explanation. Unfortunately, no coronavirus S protein fusion intermediate or pre-fusion state has been solved to date, hampering investigation of molecular interactions. We therefore analyzed the effect of variation at the 1020 position of MERS-CoV on the stability of the 6HB in the post-fusion conformation^14^. The presence of a Q1020 had previously been suggested to confer higher stability to the MERS-CoV 6HB compared to SARS-CoV^14^. Indeed, replacement of this residue results in loss of intra- and inter-helical interactions. In line with these observations, three different methods used for stability analysis were concordant in showing that the alternative arginine and histidine residues at position 1020 result in a moderate and similar level of destabilization. Although the observed ΔΔG is relatively small, it was calculated on the single monomer, and is expected to be multiplied in the trimer. The observation whereby mildly destabilizing variants are favored by selection may seem counterintuitive. Nonetheless, we show that a similar level of destabilization is observed for a mutation in MERS-Cov HR1 (T1015N) that increases infection efficiency, at least *in vitro*^31^. Mutagenesis of HR1 in retroviral type I fusion proteins has indicated that, whereas strong destabilization of the 6HB (as measured by circular dichroism) almost inevitably results in reduced infectivity, a minor stability decrease is not necessarily associated with defects in cell fusion and infection efficiency^40^,^41^,^42^. For instance, different aminoacid replacements at the same HR1 position in HIV-1 gp41 result in decreased stability, but unaffected or even increased infectivity^40^. In the case of EIAV (equine infectious anemia virus), destabilized HR1 mutants were found to display different infection phenotypes depending on temperature^42^. This observation may be extremely interesting in the context of MERS-CoV, as both bats and dromedary camels display adaptive heterothermy (i.e. sensible daily or season variation in body temperature).

Coronavirus spike proteins are highly exposed on the virus surface and represent major targets for antibody response^43^, raising the possibility that adaptive evolution of the S protein is driven by the host immune system. This hypothesis is difficult to address due to the paucity of information concerning the specific MERS-CoV epitopes recognized by human antibodies. Recently, different studies identified human antibodies againts MERS-CoV from non-immune human antibody libraries: all of them were directed against the RBD, suggesting that this region represents a major target for the host immune system^43^. Nevertheless, data on the humoral immune response to MERS-CoV in infected subjects are presently lacking, whereas such information is richer for SARS-CoV. Indeed, analysis of antibodies derived from a patient who recovered from SARS indicated that some of them recognize epitopes in the HR2 region^44^. Their neutralizing effect was ascribed to interference of the interaction between HR2 and HR1. Whether antibodies against the S2 region also arise in human subjects (or other mammalian hosts) infected with MERS-CoV and related betaCoVs remains to be determined; if this were the case, some of the selected sites we identified may be under selective pressure to evade recognition.

Finally, it is worth noting that HRs have been studied in different viruses because synthetic peptides interfering with 6HB formation are promising antiviral molecules^15^,^16^,^17^,^18^. This is also the case for MERS-CoV, and HR2-like peptides were recently shown to be effective *in vitro*^14^. These peptides were tested against a MERS-CoV strain carrying Q1020 and all include the interacting M1266 residue^14^. These antivirals may display decreased activity depending on the MERS-CoV strain and its aminoacid status at the selected 1020 position.

## Materials and Methods

### Sequences and alignments

Virus sequences were retrieved from the NCBI database and a list of accession numbers is provided as Supplementary Table S1. Sequences of Ty-BatCoV HKU4 and Pi-BatCoV HKU5 isolated in Hong Kong were derived from a previous work^28^.

Errors in the inferred multiple sequence alignment (MSA), which may be common when highly divergent sequences are analyzed, can inflate estimates of positive selection. We therefore used PRANK^45^ for building the MSA and GUIDANCE^46^ for filtering unreliably aligned codons (i.e. we masked codons with a score <0.90), as suggested^35^.

### Detection of recombination and positive selection

To detect positive selection at the *S* gene of clade c betaCoVs we applied the branch-site test from the PAML suite^22^. The test compares a model (MA) that allows positive selection on one or more lineages (foreground lineages) with a model (MA1) that does not allow such positive selection. Twice the difference of likelihood for the two models (ΔlnL) is then compared to a χ^2^ distribution with one degree of freedom^22^. Specifically, the internal branches of previously reconstructed^6^ Bayesian phylogenies of the S1 and S2 regions were set as the foreground lineages in independent tests. A false discovery rate (FDR) correction was applied to account for multiple hypothesis testing (i.e. we corrected for the number of tested branches), as suggested^47^.

Positively selected sites were identified through the BEB analysis (with a p value cutoff of 0.90), which calculates the posterior probability that each site belongs to the site class of positive selection on the foreground branch(es). Sites were validated using MEME (with the default cutoff of 0.1), which allows the distribution of dN/dS (also referred to as ω) to vary from site to site and from branch to branch at a site, therefore allowing the detection of episodic positive selection^24^.

The site models implemented in PAML were applied -independently- for the analysis of HKU4 and MERS-CoV sequences, which display very limited divergence and do not suffer from saturation problems. To detect selection, site models that allow (M2a, M8) or disallow (M1a, M7) a class of sites to evolve with ω > 1 were fitted to the data^48^. Trees were generated by maximum-likelihood using the PhyML program^49^ with a GTR model of nucleotide substitution and γ distributed rates. Positively selected sites were identified using the BEB analysis (from model M8)^50^. Again, sites were validated using MEME.

To assure consistency, all models were run using the F3 × 4 and the F61 codon frequency models.

MSAs were screened for the presence of recombination using GARD. Recombination breakpoints were considered significant if the HK (Kishino-Hasegawa) p value was <0.01.

Simultaneous inference of selection and recombination for analysis of positive selection was performed using omegaMap^29^, a program for detecting natural selection and recombination based on a model of population genetics and molecular evolution. The model uses a population genetic approximation to the coalescent with recombination. This latter is estimated from patterns of linkage disequilibrium assuming that recombination events occur only between codons and not within them. OmegaMap applies reversible-jump Markov Chain Monte Carlo (MCMC) to perform Bayesian inferences of both ω and the recombination parameter ρ, allowing both parameters to vary along the sequence. An average block length of 10 and 30 codons was used to estimate ω and ρ, respectively. To determine the influence of the choice of the priors on the posteriors, analyses were repeated with alternative sets of priors (Supplementary Table S2). Three independent omegaMap runs, each with 500,000 iterations and a 50,000 burn-in iteration, were compared to assess convergence and merged to obtain the posterior probability estimate.

The REL (random effects likelihood) analysis models variation in both dN and dS across sites according to a predefined distribution with different rate classes; positively selected sites are identified through an empirical Bayes method^51^. The default criterion of a Bayes Factor >50 was used to identify positively selected sites.

For GARD, MEME, and REL the nucleotide substitution models were chosen using a Genetic Algorithm implemented in the dataMonkey suite^52^. All analyses were performed through the DataMonkey server^53^ (http://www.datamonkey.org).

### In silico analysis of HR1 variants

The crystal structure of MERS-CoV HR1 and HR2 region was obtained from PDB (PDB ID: 4MOD).

Histidine or arginine residues were introduced at positions 1020 and suitable rotamers were sampled through the rapid torsion scan utility in Maestro (Maestro. 9.1; Schrodinger). Intraprotein interactions were calculated with PIC (protein interaction calculator)^54^.

Because programs that calculate stability changes achieve only moderate accuracy^55^, we used three different methods to assure reliability. These approaches are based on different principles. Specifically, PoPMuSiC uses statistical potentials and takes into account amino acid volume variation upon mutation^33^; FoldX uses an empirical force field and evaluates the energetic effect of point mutations and the interactions contributing to the stability of proteins^32^. Finally, I-Mutant 2.0 is based on a neural network approach to evaluate the free energy change after a single point mutation with incorporation of information on the three-dimensional structure of the protein^34^.

In FoldX and I-Mutant the ΔΔG values are calculated as follows: *ΔΔG* = *ΔG*_*mutant*_ *−* *ΔG*_*wild-type*_. In FoldX and I-Mutant ΔΔG values > 0 kcal/mol indicate mutations that decrease protein stability, whereas in PoPMuSiC ΔΔG values > 0 kcal/mol are mark of mutations increasing protein stability. Therefore, PoPMuSiC ΔΔG values were multiplied by −1 to obtain homogeneous results.

In the analysis carried out with FoldX 3D, the three-dimensional structure of the protein was repaired using the <RepairPDB> command. Mutations were introduced using the <BuildModel> command with <numberOfRuns> set to 5 and <VdWdesign> set to 0. Temperature (298K), ionic strength (0.05 M) and pH (7) were set to default values and the force-field was used to predict the water molecules on the protein surface.

## Additional Information

**How to cite this article**: Forni, D. *et al.* The heptad repeat region is a major selection target in MERS-CoV and related coronaviruses. *Sci. Rep.* **5**, 14480; doi: 10.1038/srep14480 (2015).

## Supplementary Material

Supplementary Information

## Figures and Tables

**Table 1:** Likelihood ratio test statistics for branch-site tests (clade c betaCoV).

***Spike*** **region**	**Foreground branch (MA vs MA1)**^1^	**−2ΔlnL**^2^	***p*** **value (FDR corrected** ***p*** **value)**^3^	**Sites**^4^ **identified by BEB and MEME**
S1 subunit				
	Node 18	30.92	2.69 × 10^−8^ (1.35 × 10^−7^)	R652
	Node 19	16.18	5.74 × 10^−5^ (1.43 × 10^−4^)	—
	Node 20	1.51	0.218 (0.272)	—
	Node 24	14.86	1.15 × 10^−4^ (1.92 × 10^−4^)	—
	Node 25	0.71	0.399 (0.399)	—
S2 subunit				
	Node 15	14.45	1.44 × 10^−4^ (7.20 × 10^−4^)	K854, I1180
	Node 16	12.86	3.37 × 10^−4^ (8.43 × 10^−4^)	V1060
	Node 17	5.66	0.0173 (0.0216)	M939, S1114, S1148
	Node 18	0	1 (1)	
	Node 23	7.20	7.30 × 10^−3^ (0.0121)	A1275

^1^MA and MA1 are branch-site models: MA allows a proportion of codons with dN/dS ≥ 1 on the foreground branches, whereas the MA1 model does not. The F61 codon frequency model was used.

^2^2ΔlnL is twice the difference of the natural logs of the maximum likelihood of the models being compared.

^3^Degrees of freedom = 1.

^4^Positions are relative to the MERS-CoV sequence (EMC/2012).

**Table 2:** Likelihood ratio test statistics for models of variable selective pressure among sites in MERS-CoV isolates.

***Spike* region**	**LRT model**	**Codon Frequency model**	**Degrees of freedom**	**−2ΔlnL**^3^	***p* value**	**% of sites (average dN/dS)**	**Positively selected sites**^4^ **(BEB and MEME)**
S1 subunit							
	M1a vs M2a^1^	F61	2	0.84	0.657	—	
	M7 vs M8^2^	F61	2	4.21	0.121	—	
S2 subunit							
	M1a vs M2a^1^	F61	2	7.38	0.0250	0.2 (15.83)	1020Q
	M7 vs M8^2^	F61	2	9.41	0.0091	0.2 (16.32)	1020Q

^1^M1a is a nearly neutral model that assumes one dN/dS (ω) class between 0 and 1, and one class with ω = 1; M2a (positive selection model) is the same as M1a plus an extra class of ω > 1.

^2^M7 is a null model that assumes that 0 < ω < 1 is beta distributed among sites; M8 (positive selection model) is the same as M7 but also includes an extra category of sites with ω > 1.

^3^2ΔlnL: twice the difference of the natural logs of the maximum likelihood of the models being compared.

^4^Positions are relative to the MERS-CoV sequence (EMC/2012).

## References

[b1] Zaki A. M., van Boheemen S., Bestebroer T. M., Osterhaus A. D. & Fouchier R. A. Isolation of a novel coronavirus from a man with pneumonia in Saudi Arabia. N. Engl. J. Med. 367, 1814–1820, 10.1056/NEJMoa1211721 (2012). 23075143

[b2] de Groot R. J. *et al.* Middle East respiratory syndrome coronavirus (MERS-CoV): announcement of the Coronavirus Study Group. J. Virol. 87, 7790–7792, 10.1128/JVI.01244-13 (2013). 23678167 PMC3700179

[b3] van Boheemen S. *et al.* Genomic characterization of a newly discovered coronavirus associated with acute respiratory distress syndrome in humans. MBio 3, 10.1128/mBio.00473-12 (2012). PMC3509437 23170002

[b4] Corman V. M. *et al.* Characterization of a novel betacoronavirus related to middle East respiratory syndrome coronavirus in European hedgehogs. J. Virol. 88, 717–724, 10.1128/JVI.01600-13 (2014). 24131722 PMC3911734

[b5] Tang X. C. *et al.* Prevalence and genetic diversity of coronaviruses in bats from China. J. Virol. 80, 7481–7490, doi: 10.1128/JVI.00697-06 (2006). 16840328 PMC1563713

[b6] Corman V. M. *et al.* Rooting the phylogenetic tree of middle East respiratory syndrome coronavirus by characterization of a conspecific virus from an African bat. J. Virol. 88, 11297–11303, 10.1128/JVI.01498-14 (2014). 25031349 PMC4178802

[b7] Lu G. *et al.* Molecular basis of binding between novel human coronavirus MERS-CoV and its receptor CD26. Nature 500, 227–231, 10.1038/nature12328 (2013). 23831647 PMC7095341

[b8] Yang Y. *et al.* Receptor usage and cell entry of bat coronavirus HKU4 provide insight into bat-to-human transmission of MERS coronavirus. Proc. Natl. Acad. Sci. USA. 111, 12516–12521, 10.1073/pnas.1405889111 (2014). 25114257 PMC4151778

[b9] Wang Q. *et al.* Bat origins of MERS-CoV supported by bat coronavirus HKU4 usage of human receptor CD26. Cell. Host Microbe 16, 328–337, 10.1016/j.chom.2014.08.009 (2014). 25211075 PMC7104937

[b10] Al-Tawfiq J. A. & Memish Z. A. Middle East respiratory syndrome coronavirus: transmission and phylogenetic evolution. Trends Microbiol. 22, 573–579, 10.1016/j.tim.2014.08.001 (2014). 25178651 PMC7133228

[b11] Jiang S., Lu L., Du L. & Debnath A. K. A predicted receptor-binding and critical neutralizing domain in S protein of the novel human coronavirus HCoV-EMC. J. Infect. 66, 464–466, 10.1016/j.jinf.2012.12.003 (2013). 23266463 PMC7127087

[b12] Graham R. L. & Baric R. S. Recombination, reservoirs, and the modular spike: mechanisms of coronavirus cross-species transmission. J. Virol. 84, 3134–3146, 10.1128/JVI.01394-09 (2010). 19906932 PMC2838128

[b13] Gao J. *et al.* Structure of the fusion core and inhibition of fusion by a heptad repeat peptide derived from the S protein of Middle East respiratory syndrome coronavirus. J. Virol. 87, 13134–13140, 10.1128/JVI.02433-13 (2013). 24067982 PMC3838252

[b14] Lu L. *et al.* Structure-based discovery of Middle East respiratory syndrome coronavirus fusion inhibitor. Nat. Commun. 5, 3067, 10.1038/ncomms4067 (2014). 24473083 PMC7091805

[b15] Jiang S., Lin K., Strick N. & Neurath A. R. HIV-1 inhibition by a peptide. Nature 365, 113, 10.1038/365113a0 (1993). 8371754

[b16] Watanabe S. *et al.* Functional importance of the coiled-coil of the Ebola virus glycoprotein. J. Virol. 74, 10194–10201 (2000). 11024148 10.1128/jvi.74.21.10194-10201.2000 PMC102058

[b17] Liu S. *et al.* Interaction between heptad repeat 1 and 2 regions in spike protein of SARS-associated coronavirus: implications for virus fusogenic mechanism and identification of fusion inhibitors. Lancet 363, 938–947, 10.1016/S0140-6736(04)15788-7 (2004). 15043961 PMC7140173

[b18] Bosch B. J. *et al.* Severe acute respiratory syndrome coronavirus (SARS-CoV) infection inhibition using spike protein heptad repeat-derived peptides. Proc. Natl. Acad. Sci. USA. 101, 8455–8460, 10.1073/pnas.0400576101 (2004). 15150417 PMC420415

[b19] Longdon B., Brockhurst M. A., Russell C. A., Welch J. J. & Jiggins F. M. The evolution and genetics of virus host shifts. PLoS Pathog. 10, e1004395, 10.1371/journal.ppat.1004395 (2014). 25375777 PMC4223060

[b20] Anisimova M., Nielsen R. & Yang Z. Effect of recombination on the accuracy of the likelihood method for detecting positive selection at amino acid sites. Genetics 164, 1229–1236 (2003). 12871927 10.1093/genetics/164.3.1229 PMC1462615

[b21] Kosakovsky Pond S. L., Posada D., Gravenor M. B., Woelk C. H. & Frost S. D. Automated phylogenetic detection of recombination using a genetic algorithm. Mol. Biol. Evol. 23, 1891–1901, 10.1093/molbev/msl051 (2006). 16818476

[b22] Zhang J., Nielsen R. & Yang Z. Evaluation of an improved branch-site likelihood method for detecting positive selection at the molecular level. Mol. Biol. Evol. 22, 2472–2479, 10.1093/molbev/msi237 (2005). 16107592

[b23] Gharib W. H. & Robinson-Rechavi M. The branch-site test of positive selection is surprisingly robust but lacks power under synonymous substitution saturation and variation in GC. Mol. Biol. Evol. 30, 1675–1686, 10.1093/molbev/mst062 (2013). 23558341 PMC3684852

[b24] Murrell B. *et al.* Detecting individual sites subject to episodic diversifying selection. PLoS Genet. 8, e1002764, 10.1371/journal.pgen.1002764 (2012). 22807683 PMC3395634

[b25] Rockx B. *et al.* Synthetic reconstruction of zoonotic and early human severe acute respiratory syndrome coronavirus isolates that produce fatal disease in aged mice. J. Virol. 81, 7410–7423, 10.1128/JVI.00505-07 (2007). 17507479 PMC1933338

[b26] Yamada Y., Liu X. B., Fang S. G., Tay F. P. & Liu D. X. Acquisition of cell-cell fusion activity by amino acid substitutions in spike protein determines the infectivity of a coronavirus in cultured cells. PLoS One 4, e6130, 10.1371/journal.pone.0006130 (2009). 19572016 PMC2700284

[b27] Navas-Martin S., Hingley S. T. & Weiss S. R. Murine coronavirus evolution *in vivo*: functional compensation of a detrimental amino acid substitution in the receptor binding domain of the spike glycoprotein. J. Virol. 79, 7629–7640, 10.1128/JVI.79.12.7629-7640.2005 (2005). 15919915 PMC1143675

[b28] Lau S. K. *et al.* Genetic characterization of Betacoronavirus lineage C viruses in bats reveals marked sequence divergence in the spike protein of pipistrellus bat coronavirus HKU5 in Japanese pipistrelle: implications for the origin of the novel Middle East respiratory syndrome coronavirus. J. Virol. 87, 8638–8650, 10.1128/JVI.01055-13 (2013). 23720729 PMC3719811

[b29] Wilson D. J. & McVean G. Estimating diversifying selection and functional constraint in the presence of recombination. Genetics 172, 1411–1425, 10.1534/genetics.105.044917 (2006). 16387887 PMC1456295

[b30] Cotten M. *et al.* Spread, circulation, and evolution of the Middle East respiratory syndrome coronavirus. MBio 5, 10.1128/mBio.01062-13 (2014). PMC3944817 24549846

[b31] Scobey T. *et al.* Reverse genetics with a full-length infectious cDNA of the Middle East respiratory syndrome coronavirus. Proc. Natl. Acad. Sci. USA. 110, 16157–16162, 10.1073/pnas.1311542110 (2013). 24043791 PMC3791741

[b32] Schymkowitz J. *et al.* The FoldX web server: an online force field. Nucleic Acids Res. 33, W382–8, 10.1093/nar/gki387 (2005). 15980494 PMC1160148

[b33] Dehouck Y., Kwasigroch J. M., Gilis D. & Rooman M. PoPMuSiC 2.1: a web server for the estimation of protein stability changes upon mutation and sequence optimality. BMC Bioinformatics 12, 151-2105-12-151, 10.1186/1471-2105-12-151 (2011). PMC3113940 21569468

[b34] Capriotti E., Fariselli P. & Casadio R. I-Mutant2.0: predicting stability changes upon mutation from the protein sequence or structure. Nucleic Acids Res. 33, W306–10, 10.1093/nar/gki375 (2005). 15980478 PMC1160136

[b35] Privman E., Penn O. & Pupko T. Improving the performance of positive selection inference by filtering unreliable alignment regions. Mol. Biol. Evol. 29, 1–5, 10.1093/molbev/msr177 (2012). 21772063

[b36] de Haan C. A. *et al.* Cooperative involvement of the S1 and S2 subunits of the murine coronavirus spike protein in receptor binding and extended host range. J. Virol. 80, 10909–10918, 10.1128/JVI.00950-06 (2006). 16956938 PMC1642182

[b37] McRoy W. C. & Baric R. S. Amino acid substitutions in the S2 subunit of mouse hepatitis virus variant V51 encode determinants of host range expansion. J. Virol. 82, 1414–1424, 10.1128/JVI.01674-07 (2008). 18032498 PMC2224421

[b38] Pacheco B., Basmaciogullari S., Labonte J. A., Xiang S. H. & Sodroski J. Adaptation of the human immunodeficiency virus type 1 envelope glycoproteins to new world monkey receptors. J. Virol. 82, 346–357, 10.1128/JVI.01299-07 (2008). 17959679 PMC2224371

[b39] Mori K., Rosenzweig M. & Desrosiers R. C. Mechanisms for adaptation of simian immunodeficiency virus to replication in alveolar macrophages. J. Virol. 74, 10852–10859 (2000). 11044136 10.1128/jvi.74.22.10852-10859.2000 PMC110966

[b40] Eggink D. *et al.* Detailed mechanistic insights into HIV-1 sensitivity to three generations of fusion inhibitors. J. Biol. Chem. 284, 26941–26950, 10.1074/jbc.M109.004416 (2009). 19617355 PMC2785381

[b41] Suntoke T. R. & Chan D. C. The fusion activity of HIV-1 gp41 depends on interhelical interactions. J. Biol. Chem. 280, 19852–19857, 10.1074/jbc.M502196200 (2005). 15772068

[b42] Du J. *et al.* Structural and biochemical insights into the V/I505T mutation found in the EIAV gp45 vaccine strain. Retrovirology 11, 26-4690-11-26, 10.1186/1742-4690-11-26 (2014). PMC3997929 24656154

[b43] Ying T., Li H., Lu L., Dimitrov D. S. & Jiang S. Development of human neutralizing monoclonal antibodies for prevention and therapy of MERS-CoV infections. Microbes Infect. 17, 142–148, 10.1016/j.micinf.2014.11.008 (2015). 25456101 PMC4308519

[b44] Rockx B. *et al.* Structural basis for potent cross-neutralizing human monoclonal antibody protection against lethal human and zoonotic severe acute respiratory syndrome coronavirus challenge. J. Virol. 82, 3220–3235, 10.1128/JVI.02377-07 (2008). 18199635 PMC2268459

[b45] Loytynoja A. & Goldman N. An algorithm for progressive multiple alignment of sequences with insertions. Proc. Natl. Acad. Sci. USA. 102, 10557–10562, 10.1073/pnas.0409137102 (2005). 16000407 PMC1180752

[b46] Penn O. *et al.* GUIDANCE: a web server for assessing alignment confidence scores. Nucleic Acids Res. 38, W23–8, 10.1093/nar/gkq443 (2010). 20497997 PMC2896199

[b47] Anisimova M. & Yang Z. Multiple hypothesis testing to detect lineages under positive selection that affects only a few sites. Mol. Biol. Evol. 24, 1219–1228, 10.1093/molbev/msm042 (2007). 17339634

[b48] Yang Z. PAML 4: phylogenetic analysis by maximum likelihood. Mol. Biol. Evol. 24, 1586–1591, 10.1093/molbev/msm088 (2007). 17483113

[b49] Guindon S., Delsuc F., Dufayard J. F. & Gascuel O. Estimating maximum likelihood phylogenies with PhyML. Methods Mol. Biol. 537, 113–137, 10.1007/978-1-59745-251-9_6 (2009). 19378142

[b50] Anisimova M., Bielawski J. P. & Yang Z. Accuracy and power of bayes prediction of amino acid sites under positive selection. Mol. Biol. Evol. 19, 950–958 (2002). 12032251 10.1093/oxfordjournals.molbev.a004152

[b51] Kosakovsky Pond S. L. & Frost S. D. Not so different after all: a comparison of methods for detecting amino acid sites under selection. Mol. Biol. Evol. 22, 1208–1222, 10.1093/molbev/msi105 (2005). 15703242

[b52] Delport W. *et al.* CodonTest: modeling amino acid substitution preferences in coding sequences. PLoS Comput. Biol. 6, 10.1371/journal.pcbi.1000885 (2010). PMC2924240 20808876

[b53] Delport W., Poon A. F., Frost S. D. & Kosakovsky Pond S. L. Datamonkey 2010: a suite of phylogenetic analysis tools for evolutionary biology. Bioinformatics 26, 2455–2457, 10.1093/bioinformatics/btq429 (2010). 20671151 PMC2944195

[b54] Tina K. G., Bhadra R. & Srinivasan N. PIC: Protein Interactions Calculator. Nucleic Acids Res. 35, W473–6, 10.1093/nar/gkm423 (2007). 17584791 PMC1933215

[b55] Khan S. & Vihinen M. Performance of protein stability predictors. Hum. Mutat. 31, 675–684, 10.1002/humu.21242 (2010). 20232415

[b56] Millet J. K. & Whittaker G. R. Host cell entry of Middle East respiratory syndrome coronavirus after two-step, furin-mediated activation of the spike protein. Proc. Natl. Acad. Sci. USA. 111, 15214–15219, 10.1073/pnas.1407087111 (2010). 25288733 PMC4210292

