# Supplementary material for: The heptad repeat region is a major selection target in MERS-CoV and related coronaviruses
Source: Sci Rep. 2015 Sep 25;5:14480. doi: 10.1038/srep14480 (PMC4585914; doi:10.1038/srep14480)

The heptad repeat region is a major selection target in MERS-CoV and related coronaviruses

Diego Forni, Giulia Filippi, Rachele Cagliani, Luca De Gioia, Uberto Pozzoli, Nasser Al-Daghri, Mario Clerici, Manuela Sironi

Supplementary Information.

Supplementary Table S1. Strains and accession numbers of analyzed coronaviruses.

Supplementary Table S2. Parameters used for omegaMap analysis.

Supplementary Table S3. Likelihood ratio test statistics for branch-site tests (clade c betaCoV).

Supplementary Table S4. Likelihood ratio test statistics for models of variable selective pressure among sites for Ty-batCoV HKU4 S gene alignment.

Supplementary Table S5. Positively selected sites identified in Pi-BatCoV HKU5 spike gene.

Supplementary Table S6. Likelihood ratio test statistics for models of variable selective pressure among sites in MERS-CoV isolates.

Supplementary Figure S1. Plot of the posterior probability of ρ for Pi-BatCoV HKU5 sequences.

Supplementary Table S1. Strains and accession numbers of analyzed coronaviruses.

Strain name	Accession number	Origin	Analysis performed
EriCoV/2012-174	KC545383	Hedgehog	Clade c betaCoV, branch-site test
EriCoV/2012-216	KC545386	Hedgehog	Clade c betaCoV, branch-site test
NeoCoV	KC869678	Bat	Clade c betaCoV, branch-site test
BtCoV/KW2E-F93	JX899383	Bat	Clade c betaCoV, branch-site test
BtCoV/133	NC_008315	Bat	Clade c betaCoV, branch-site test
HKU4-1	NC_009019	Bat	Clade c betaCoV, branch-site test
HKU5-5	EF065512	Bat	Clade c betaCoV, branch-site test
HKU5-1	NC_009020	Bat	Clade c betaCoV, branch-site test
Al-Hasa 1 2013	KF186567	Human	Clade c betaCoV, branch-site test; MERS-CoV, site model test
Al-Hasa 11c 2013	KF600636	Human	MERS-CoV, site model test
Al-Hasa 12 2013	KF600627	Human	MERS-CoV, site model test
Al-Hasa 14b 2013	KF600643	Human	MERS-CoV, site model test
Al-Hasa 15 2013	KF600645	Human	MERS-CoV, site model test
Al-Hasa 16 2013	KF600644	Human	MERS-CoV, site model test
Al-Hasa 17 2013	KF600647	Human	MERS-CoV, site model test
Al-Hasa 18 2013	KF600651	Human	MERS-CoV, site model test
Al-Hasa 19 2013	KF600632	Human	MERS-CoV, site model test
Al-Hasa 2 2013	KF186566	Human	MERS-CoV, site model test
Al-Hasa 21 2013	KF600634	Human	MERS-CoV, site model test
Al-Hasa 25 2013	KJ156866	Human	MERS-CoV, site model test
Al-Hasa 3 2013	KF186565	Human	MERS-CoV, site model test
Al-Hasa 4 2013	KF186564	Human	MERS-CoV, site model test
Al-Hasa 7b 2013	KF600623	Human	MERS-CoV, site model test
England 1	KC164505	Human	Clade c betaCoV, branch-site test; MERS-CoV, site model test
England/2/2013	KM015348	Human	MERS-CoV, site model test
Bisha 1 2012	KF600620	Human	MERS-CoV, site model test
Buraidah 1 2013	KF600630	Human	MERS-CoV, site model test
Camel/Qatar 2 2014	KJ650098	Camel	MERS-CoV, site model test
EMC/2012	JX869059	Human	Clade c betaCoV, branch-site test; MERS-CoV, site model test
Florida/USA-2 Saudi Arabia 2014	KJ829365	Human	MERS-CoV, site model test
FRA/UAE	KF745068	Human	MERS-CoV, site model test
Hafr-Al-Batin 1 2013	KF600628	Human	MERS-CoV, site model test
Hafr-Al-Batin 2 2013	KJ156910	Human	MERS-CoV, site model test
Hafr-Al-Batin 6 2013	KJ156874	Human	MERS-CoV, site model test
Indiana/USA-1 Saudi Arabia 2014	KJ813439	Human	MERS-CoV, site model test
Jeddah 1 2013	KJ556336	Human	MERS-CoV, site model test

Jordan-N3/2012	KC776174	Human	MERS-CoV, site model test
KFU-HKU 1	KJ650297	Camel	MERS-CoV, site model test
KFU-HKU 19Dam	KJ650296	Camel	MERS-CoV, site model test
KFU-KHU 13	KJ650295	Camel	MERS-CoV, site model test
KSA-Camel-363	KJ713298	Camel	MERS-CoV, site model test
KSA-Camel-376	KJ713299	Camel	MERS-CoV, site model test
KSA-Camel-378	KJ713296	Camel	MERS-CoV, site model test
KSA-Camel-503	KJ713297	Camel	MERS-CoV, site model test
KSA-Camel-505	KJ713295	Camel	MERS-CoV, site model test
Madinah 3b 2013	KJ156916	Human	MERS-CoV, site model test
MERS-CoV-Jeddah-Camel-1	KF917527	Camel	MERS-CoV, site model test
MERS-CoV-Jeddah-human-1	KF958702	Human	MERS-CoV, site model test
Munich	KF192507	Human	MERS-CoV, site model test
NRCE-HKU205	KJ477102	Camel	Clade c betaCoV, branch-site test; MERS-CoV, site model test
Qatar 3	KF961221	Human	MERS-CoV, site model test
Qatar 4	KF961222	Human	MERS-CoV, site model test
Riyadh 1 2012	KF600612	Human	Clade c betaCoV, branch-site test; MERS-CoV, site model test
Riyadh 14 2013	KJ156934	Human	MERS-CoV, site model test
Riyadh 2 2012	KF600652	Human	MERS-CoV, site model test
Riyadh 3 2013	KF600613	Human	MERS-CoV, site model test
Riyadh 4 2013	KJ156952	Human	MERS-CoV, site model test
Riyadh 5 2013	KJ156944	Human	MERS-CoV, site model test
Riyadh 8b 2013	KJ156942	Human	MERS-CoV, site model test
Riyadh 9 2013	KJ156869	Human	MERS-CoV, site model test
Taif 1 2013	KJ156949	Human	MERS-CoV, site model test
Wadi-Ad-Dawasir 1 2013	KJ156881	Human	MERS-CoV, site model test

Supplementary Table S2. Parameters used for omegaMap analysis.

Parameter	Prior A	Prior B
μ (synonymous transversion)	Improper inverse	Exponential (mean 0.07)
κ (transition/transversion rate)	Improper inverse	Exponential (mean 3.0)
ϕ (insertion/deletion rate)	Improper inverse	Exponential (mean 0.1)
ω (dN/dS)	Inverse (range 0.01-100)	Exponential (mean 1.0)
ρ (recombination)	Inverse (range 0.01-100)	Exponential (mean 0.1)
omegaBlok	10	10
rhoBlock	30	30
Thinning	100	100
Iterations	500,000	500,000
Burn-in	50,000	50,000

Supplementary Table S3. Likelihood ratio test statistics for branch-site tests (clade c betaCoV).

***Spike* region**	**Foreground branch (MA vs MA1)[1]**	**-2ΔlnL[2]**	**Degrees of freedom**	***p* value (FDR corrected *q* value)**
**S1 *SUBUNIT***				
	Node 18	13.48	1	$2.41x10^{-4}$ ($1.21x10^{-3}$)
	Node 19	11.94	1	$5.46x10^{-4}$ ($1.38x10^{-3}$)
	Node 20	0.98	1	0.325 (0.325)
	Node 24	6.73	1	$9.46x10^{-3}$ (0.0158)
	Node 25	5.85	1	0.0156 (0.0195)
**S2 *SUBUNIT***				
	Node 15	7.29	1	$6.92x10^{-3}$ (0.0108)
	Node 16	16.38	1	$5.18x10^{-5}$ ($2.59x10^{-4}$)
	Node 17	6.89	1	$8.67x10^{-3}$(0.0108)
	Node 18	0	1	1 (1)
	Node 23	7.20	1	$7.30x10^{-3}$ (0.0108)

[1] MA and MA1 are branch-site models that assume four classes of sites: the MA model allows a proportion of codons to have ω≥ 1 on the foreground branches, whereas the MA1 model does not. The F3x4 codon frequency model was used.
[2] 2ΔlnL is twice the difference of the natural logs of the maximum likelihood of the models being compared.

Supplementary Table S4. Likelihood ratio test (LRT) statistics for models of variable selective pressure among sites for Ty-batCoV HKU4 *S* gene alignment.

LRT model	Codon Frequency model	Degrees of freedom	-2ΔlnL[3]	*p* value
M1a vs M2a[1]	F3x4	2	0.75	0.686
	F61	2	1.18	0.552
M7 vs M8[2]	F3x4	2	0.92	0.63
	F61	2	0.77	0.681

[1] M1a is a nearly neutral model that assumes one ω class between 0 and 1, and one class with ω=1; M2a (positive selection model) is the same as M1a plus an extra class of ω >1.
[2] M7 is a null model that assumes that 0<ω<1 is beta distributed among sites; M8 (positive selection model) is the same as M7 but also includes an extra category of sites with ω>1.
[3] 2ΔlnL: twice the difference of the natural logs of the maximum likelihood of the models being compared.

Supplementary Table S5. Positively selected sites identified in Pi-BatCoV HKU5 *spike* gene. REL (Random Effects Likelihood) was run accounting for the GARD-estimated phylogenies (i.e. by accounting for recombination).

Method	N. of isolates	Positively selected sites[1]
REL	15	N131, S361, Q389, T514

[1] positions refer to HKU5-1 strain.

Supplementary Table S6. Likelihood ratio test statistics for models of variable selective pressure among sites in MERS-CoV isolates.

Spike region	LRT model	Codon Frequency model	Degrees of freedom	-2ΔlnL[3]	*p* value	% of sites (average dN/dS)
**S1 subunit**						
	M1a vs M2a[1]	F3x4	2	0.24	0.887	-
	M7 vs M8[2]	F3x4	2	0.44	0.804	-
**S2 subunit**						
	M1a vs M2a[1]	F3x4	2	7.57	0.023	0.2 (29.96)
	M7 vs M8[2]	F3x4	2	7.03	0.0297	0.2 (31.96)

[1] M1a is a nearly neutral model that assumes one dN/dS (ω) class between 0 and 1, and one class with ω=1; M2a (positive selection model) is the same as M1a plus an extra class of ω >1.
[2] M7 is a null model that assumes that 0<ω<1 is beta distributed among sites; M8 (positive selection model) is the same as M7 but also includes an extra category of sites with ω>1.
[3] 2ΔlnL: twice the difference of the natural logs of the maximum likelihood of the models being compared.
[4] Positions are relative to the MERS-CoV sequence (EMC/2012).

Supplementary Figure S1. Plot of the posterior probability of ρ (recombination parameter) for Pi-BatCoV HKU5 sequences.
Analysis was repeated with alternative sets of priors and three independent omegaMap runs, each with 500,000 iterations and a 50,000 iteration burn-in, were compared to assess convergence and merged to obtain the posterior probability. Solid line: mean; gray shading: 95% highest posterior density interval.

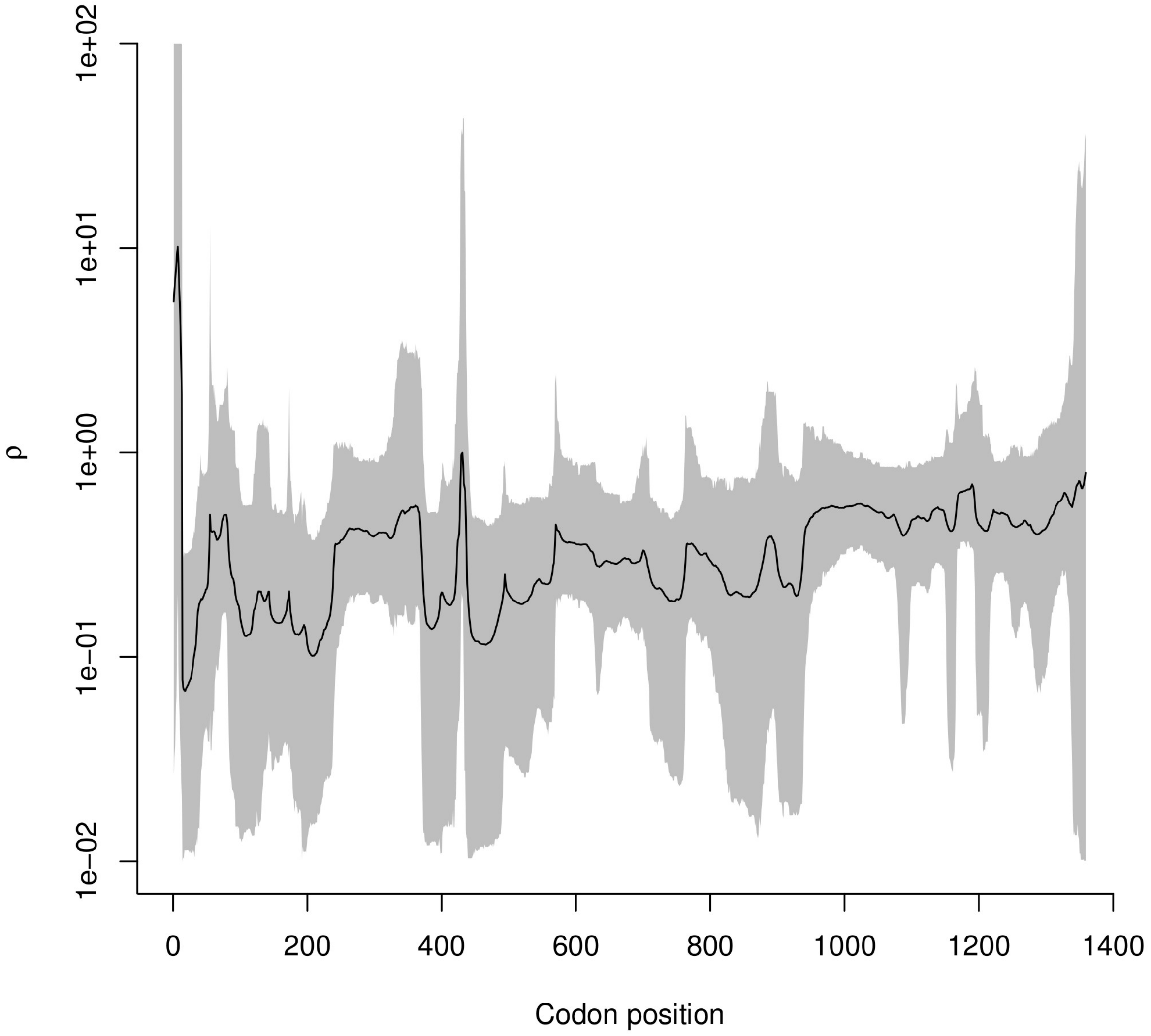

Supplement: Supplementary Information [file srep14480-s1.pdf]
